# Phubbing behavior, personality, and use of instagram by Brazilian adults: a correlational and predictive study

**DOI:** 10.1186/s41155-023-00268-w

**Published:** 2023-09-04

**Authors:** Adriane de Almeida Santos, Breno de Oliveira Ferreira, Consuelena Lopes Leitão, Iolete Ribeiro da Silva, Marck de Souza Torres

**Affiliations:** Federal University of Amazonas, Av. Rodrigo Otávio, 6200 - Setor Sul - Campus Universitário - Bloco X Coroado - CEP - 69080-900, Manaus, AM Brazil

**Keywords:** Phubbing behavior, Personality, Big five, Instagram

## Abstract

Phubbing behavior is a phenomenon that consists in ignoring people in situations of social interaction whilst paying attention to one’s smartphone. The study of this behavior enables reflection on the development of healthy behavior patterns when using technology and the design of intervention strategies to cope with phubbing behavior. This study aimed to investigate the relationship between phubbing behavior, use of Instagram, personality traits (Big Five), and sociodemographic variables (gender, education, and age) among Brazilian adults. This was a cross-sectional study with a sample of 1551 adults (61.7% women; 29.9% men), aged between 18 and 76 years (M = 31.6 years; SD = 9.6 years). The results of the correlation analysis indicated that excessive use of Instagram showed a high, positive association ρ (1551) = 0.442 with Phubbing Behavior and a moderate one with neuroticism ρ (1551) = 0.272. Phubbing behavior was positively and moderately with neuroticism ρ (1551) = 0.290, but it had a weak, negative correlation with age ρ (1551) = -0.117; *p* < 0.001. Multiple linear regression analysis (forward method) indicated that the variables that most strongly impacted Phubbing Behavior were neuroticism (ΔR2 = .236), conscientiousness (ΔR2 = .244) and use of Instagram (ΔR2 = .204). This result indicates that conscientiousness may have a predictive potential to decrease phubbing behavior, whereas neuroticism and use of Instagram may lead to increased phubbing. Multivariate Analysis of Variance indicated that excessive use of Instagram registered higher scores for women (M = 11.48; SD = 0.21) than for men (M = 9.45; SD = 0.27, *p* < 0.001). It was concluded that while conscientiousness can function as a protective factor for the development of phubbing behavior, high levels of neuroticism and excessive use of Instagram have greater potential to act as risk factors for it. In addition, neuroticism is also a risk factor for excessive use of Instagram, and women are more prone to such overuse.

## Introduction

Online social networks (OSNs) are deeply integrated into people's daily lives. Over the past few decades, the use of OSNs such as Instagram has grown rapidly. The Digital Global Overview Report published in April 2023, pointed out that Instagram has 1.63 billion users, which accounts for 20.3% of the global population. Brazil has the third largest Instagram audience, with 132.5 million users, only preceded by the USA and India (We Are Social, 2023). This popularity has raised awareness about the risks of using Instagram, such as impacts on mental health (Foroughi et al., 2022) and the development of problematic technology-related behaviors — for example, phubbing behavior (Balta et al., 2020; van der Schyff et al., 2022).

Phubbing behavior (phone + snubbing) is the act of ignoring one or more people in social situations to devote attention to one’s smartphone, avoiding face-to-face interpersonal communication. A term has been coined for practitioners of this behavior: “phubbers”, while those who are ignored are referred to as “phubbees”. When the Phubbing Scale was developed to measure this behavior, two factors were identified: communication disorder (i.e., frequency with which individuals disturb their face-to-face communications by using their cell phones) and phone obsession (i.e., need for one’s cell phone in environments that do not involve face-to-face interaction). Phubbing behavior is related to addiction to cell phones/smartphones, the Internet, and digital games, but the association with addiction to OSNs was what stood out the most. Instagram is an example of an OSN created specifically to be used through an application on smartphones. Although it can be accessed through a computer, few functions are available on the web version, i.e., its features are fully usable only when it is accessed through a smartphone (Blanca & Bendayan, 2018; Abeele, 2020; Chotpitayasunondh & Douglas, 2018; Karadağ et al., 2015).

The relationship between Instagram use and phubbing behavior has been investigated, and results confirm the existence of a positive correlation between them (Balta et al., 2020; Phang et al., 2020; Van der Schyff et al., 2022). In an attempt to better understand the development and characteristics of this phenomenon, the role of personality needs to be taken into consideration. To better understand the relationships between personality traits and technological variables, such as smartphone use (Pancani et al., 2020), internet dependency (Kuang et al., 2020), and online social networks (Whaite et al., 2018), researchers have been investigating them for some time. Understanding personality traits is key for understanding different ways in which people relate to technology and develop unhealthy behaviors such as phubbing (Kircaburun & Griffiths, 2018).

The Big Five model identifies five personality factors: Neuroticism, Extraversion, Agreeableness, Conscientiousness, and Openness to experience. Individuals are characterized by having these factors to different extents, i.e., with greater or lesser incidence of each factor, alternating between high and low poles along a spectrum. Neuroticism is the factor most closely associated with emotional characteristics; it refers to the level of adjustment and emotional instability of individuals. Extraversion is related to people's sociability, indicating how communicative, active, and assertive they are. Agreeableness refers to the quality of interpersonal relationships and prosocial behavior of individuals. Conscientiousness organizes and directs behavior. Finally, Openness to experience refers to exploratory behaviors and recognition of the importance of having new experiences and the need for variety of ideas, creativity, intellectual interests, different emotions, and aesthetic sensitivity (Costa & McCrae, 1992).

Instagram has also appeared as a mediator between phubbing behavior and neuroticism; this personality trait correlated positively with excessive use of Instagram. The latter, in turn, correlated positively with higher rates of phubbing behavior, which indicated that neurotic individuals use Instagram more excessively and, consequently, exhibit a higher level of phubbing behavior (Balta et al., 2020). Notwithstanding, the Turkish study only investigated the relationship between phubbing behavior, Instagram, and neuroticism, without taking the other Big Five personality traits into account.

Concerning the relationship between personality traits and Instagram use, Kircaburun and Griffiths (2018) conducted a study with young Turkish individuals and found that amiability and conscientiousness were negatively correlated with Instagram addiction. Moore and Craciun’s study (2021), carried out in the USA, found that extroversion was related to a larger number of posts and a higher tendency to depend on the said network, while the Polish and Turkish researchers indicated that neuroticism was positively associated with excessive use of Instagram (Drążkowski et al., 2022; Balta et al., 2020).

When addressing the relationship between the Big Five personality traits and phubbing behavior, the studies carried out by Çikrikci et al. (2019) and Erzen et al. (2021) with Turkish university students had the same outcomes, which showed that neuroticism correlated positively with phubbing behavior. Moreover, while Erzen et al. (2021) reported a significant correlation between neuroticism and the phubbing behavior known as Communication Disturbance factor, the study of Guazzini et al. (2019) with Italian subjects discovered that neuroticism correlated positively with the phubbing behavior referred to as mobile phone obsession factor. It was then concluded that personality traits have an augmentative effect on the obsessive component.

Different researchers have investigated the relationships between phubbing behavior, Instagram use and gender, age, and education variables. Karadağ et al., (2015) found that phubbing rates are higher in women than in men. A negative and low relationship between age and phubbing was found by İliç and Tanyeri (2021). This result was confirmed by Błachnio et al., (2021), whose study showed that the phone obsession factor was negatively related to age.

Balta et al. (2020) found that when compared to men, women had higher levels of problematic Instagram use. Rozgonjuk et al., (2020a, 2020b) found a negative relationship between age and Instagram use. As for education, Van der Schyff et al. (2022) indicated that an individual’s educational level is positively and significantly related to excessive Instagram use. Parmaksız and Kılıçarslan (2021) found a positive relationship with education considering elementary, secondary, and undergraduate levels, but there was no significant relationship between phubbing and graduate education. These results suggest that there is a profile of subjects who are more susceptible to the development of problematic behavior patterns when using technology. Therefore, further research is needed on the afore-mentioned variables, as it can not only provide further insights into how this phenomenon manifests itself in society and in individuals, but also enable the design of prevention and intervention measures.

Although previous studies have addressed the possible relation between psychological, behavioral, and technological variables, there is still little research, especially in Brazil, on the relationship between Instagram, phubbing behavior, and the personality traits from the Big Five model. In addition, this behavior is closely linked to the use of smartphones and OSNs, and there is an intrinsic relationship between Instagram and smartphones. For this reason, it is worth considering the relationship between Instagram and phubbing behavior and the role played by personality in the development of this relationship. Therefore, the present study is relevant not only at the national level, but also internationally, since Brazil has a very large number of Instagram users worldwide. Findings about this population can help researchers reflect upon this theme and create new concepts while conducting cyberpsychology studies around the world.

Thus, the present study aims to investigate the relationship between phubbing behavior, Instagram use, and personality, considering the Big Five model personality traits and sociodemographic variables of gender, education, and age among Brazilian adults.

## Method

### Study design and participants

The present study followed a cross-sectional predictive correlational design, based on a quantitative methodological study. The sample was composed of a total of 1,551 adults: 65.5% cisgender females, 31.8% cisgender males, 1.4% non-binary individuals, 0.8% transgender women and 0.5% transgender men, ranging in age from 18 to 76 years old (M = 31.6 years old; SD = 9.6 years old). They participated as anonymous volunteers and did not receive any financial compensation for their contribution to this study. Among the participants, 62.5% were white and 25.8% were of mixed race; 44.3% had a master’s degree/PhD while 19% did not finish higher education. Participants were from the five different regions of Brazil: 38.9% from the Southeast; 28% from the South; 15.5% from the Northeast; 11.3% from the North; 5.2% from the Central-West; and 1.1 from the Federal District (where the country’s capital, Brasília, is located). The following inclusion criteria were adopted: being 18 years old or over, being a Brazilian citizen, and residing in any Brazilian state or in the Federal District.

### Variables and instruments

#### Sociodemographic data and Instagram time usage

A questionnaire was designed to collect data from the participants, namely information about sociodemographic characteristics, such as age, localization, gender, sexual orientation, skin color, education, and questions about frequency of Instagram use.

#### Phubbing behavior

To assess the levels of phubbing behavior in the participants, a 9-item Brazilian Portuguese version for the Phubbing Scale (PS) was used. It was adapted from the version developed by Karadağ et al. (2015) and validated psychometrically by Santos (2023) to be used with the Brazilian population. The PS is answered by means of an adapted five-point *Likert* scale (1 = never to 5 = always). The PS assesses two factors: (1) Communication disturbance: higher scores indicate that ongoing communication among participants is usually hindered when they use their mobile phones in a face-to-face communication environment. This factor is formed by items 1, 2, 3, and 4. (2) Mobile phone obsession: higher scores indicate that participants constantly need their mobile phones in environments without face-to-face communication. This factor is formed by items 5, 6, 7, 8, and 9. In the study for validating the scale for the Brazilian population, Cronbach’s Alpha values were: 0.83 for factor (1) Communication disturbance; 0.75 for factor (2) Mobile phone obsession, and the general alpha for the Brazilian version of the PS is 0.83 (Santos, 2023).

#### Personality according to the Big Five model

Personality traits based on the Big Five theoretical model were assessed using the Short Form Scale of Descriptors of the Five Personality Factors (RED5), developed and validated by Natividade and Hutz (2015) for the Brazilian population. This instrument is made up of twenty items, four per factor, that is, adjectives or small expressions, for example: “communicative”, “emotionally stable”, “curious”. On a 7-score *Likert* scale, respondents were asked how much they agree that the adjective or expression accurately describes them, with scores ranging from 1—totally disagree—to 7—totally agree. The closer to seven the means of the factors are, the higher the intensity of the traits. Natividade and Hutz (2015) found alpha coefficients between 0.59 and 0.84 for the factors and test–retest correlation from 0.69 to 0.81.

In RED5, Neuroticism, which refers to a tendency to demonstrate emotional instability, to experience negative emotions, anxiety, and depression, is composed of the adjectives anxious, calm, temperamental, and by the expression “emotionally stable”. Extroversion, a tendency to seek stimulation in interacting with others, to be active and communicative, is composed of the adjectives: extrovert; communicative; shy and quiet. Agreeableness, which refers to a tendency to show empathy, altruism, and a prosocial behavior, is composed of the adjectives: unfriendly, sympathetic, hard-hearted, and friendly. Conscientiousness, that is, a tendency to self-control in carrying out tasks that lead to a goal, to be disciplined and organized, is composed of the adjectives: undisciplined, responsible, disorganized, and hardworking. Finally, Openness to experience, a tendency to try new things, to demonstrate curiosity and intellectual complexity, is formed by the expressions: “dislikes change”, “open to new experiences” and by the adjectives “conventional” and “curious” (John et al., 2010; Nunes et al., 2009; Natividade & Hutz, 2015).

#### Instagram use

Research on Instagram use has been conducted using the Bergen Instagram Addiction Scale (BIAS), which, originally, was based on the Bergen Facebook Addiction Scale (BFAS) (Andreassen et al., 2012). It was first adapted to a Brazilian context by Monteiro et al. (2020), who changed its name to Bergen Instagram Addiction Scale (BIAS). BIAS is made up of six items, answered in the form of the five-score Likert Scale (1 – Very Rarely; 5 – Very Often). These items address Instagram use and how people relate to it during a 1-year period (e.g., Item 4: In the last year, have you tried to cut down the amount of time you spend on Instagram and failed?).

#### Procedures

All participants were included in this study after reading and signing an Informed Consent Form (ICF). A self-administered questionnaire using the Google Forms tool, which enables the creation and application of online surveys, was used for collecting data. The questionnaire was shared via social networks (e.g.: Facebook, WhatsApp, E-mail, Instagram) by means of a hyperlink plus text that described the objective of the research and the target population. The application of the instruments took about 15 min. By clicking on the hyperlink, the participants were taken to a page containing the ICF. They were required to fill in the ICF before they were given access to the questionnaire. Data collection was conducted from June to August 2022.

#### Data analysis

For the present study, correlation analyses were performed to verify the variance between the Phubbing Scale and the Bergen Instagram Addiction Scale (BIAS), the Big Five and sociodemographic variables (e.g., age, education, and gender). Then, in order to estimate a predictive model for phubbing behavior, multiple linear regression analysis was performed using the forward method, based on the choice of a set of possible predictive variables. When running the analysis, instead of starting with all the variables in the model, the process started with an “empty model”, also known as a Null Model. Next, predictor variables were automatically added to the model, one at a time, starting with the one with the lowest *p*-value. This process continued until all selected predictor variables were tested, and those that do not reach a predefined *p*-value threshold (usually with p above 0.05) were excluded from the model. Finally, Multivariate Analysis of Variance (MANOVA) was performed for an analysis of the relationship between phubbing behavior and Instagram Use considering the different levels of education of men and women in the sample. The respective analyses were estimated using a pattern with 1,000 re-samplings in the non-parametric bootstrap, with a 95% bias-corrected and accelerated (BCa) confidence interval. The Statistical Package for Social Sciences (version 20.0) was used (SPSS ® Inc, Chicago, IL).

## Results

The results of the correlation analysis for phubbing indicated that excessive use of Instagram has a positive and strong correlation ρ (1551) = 0.442; *p* < 0.001, while Neuroticism has a positive and moderate correlation ρ (1551) = 0.290; *p* < 0.001). Two other variables showed negative and weak correlations, namely age ρ (1551) = -0.117; *p* < 0.001 and Conscientiousness ρ (1551) = -0.185; *p* < 0.001. As for Instagram, the results indicated that, in addition to the positive and strong correlation ρ (1551) = 0.442; *p* < 0.001 with phubbing behavior, age showed a moderate negative correlation ρ (1551) = -0.250; *p* < 0.001; gender, a weak negative correlation ρ (1551) = -0.193; *p* < 0.001; and Neuroticism, a moderate positive correlation ρ (1551) = 0.272; *p* < 0.001 Table 1.

**Table 1:** Analysis of correlation between variables

	Phubbing	Ebai
Phubbing	1.000	.442^**^
Education	-.061^*^	-.064^*^
Age	-.117^**^	-.250^**^
Gender	-.049	-.193^**^
Extraversion	.017	.045
Agreeableness	-.054^*^	.016
Neuroticism	.290^**^	.272^**^
Conscientiousness	-.185^**^	-.088^**^
Openness to Experience	-.011	-.010
BIAS	.442^**^	1.000

^*^ = *p* < 0.05; ** = *p* < 0.01. *BIAS* Bergen Instagram Addiction Scale

The results of the multiple linear regression analysis (forward method) demonstrated the influence of Neuroticism, Conscientiousness, and excessive use of Instagram on phubbing behavior (*F* (3, 1547) = 167.844, *p* < 0.001; R^2^adjusted = 0.244). Table 2 shows the coefficients for all significant predictors. As can be seen, the variable with the greatest potential to decrease phubbing behavior was Conscientiousness, which accounts for 24% of the outcome.

**Table 2:** Predictive variables of phubbing behavior

Predictors	Standardized Coefficients	*t*	Sig	*R*^*2*^	*ΔR*^*2*^
	*Beta*				
(Constant)	-	20.128	0.000	-	-
Neuroticism	.163	6.919	0.000	.237	.236
Conscientiousness	-.095	-4.162	0.000	.246	.244
BIAS	.399	17.408	0.000	.205	.204

*BIAS* Bergen Instagram Addiction Scale

The BOX’s M test took into account the assumption of homogeneity of covariance (BOX´S *M* = 37.982; *F*(27, 284,408.671), *p* = 0.085). The MANOVA results showed that there was no relevant effect of school background (*F* (8, 3002.00) = 2.091, *p* = 0.033; *h*^2^ = 0.006), neither regarding the interaction between gender and education (*F*(2, 3002,00) = 0.815, *p* = 0.589; *h*^2^ = 0.002). Only the gender variable presented statistically significant results, with an average effect (*F*(2, 1500) = 17.502. *p* < 0.001; *h*^2^ = 0.23). Table 3 shows the descriptive statistics of all groups.

**Table 3:** MANOVA for the BIAS and phubbing variables subdivided by education and gender

	GENDER	EDUCATION	Average	SD
BIAS	Females	1	11.13	5.26
		2	12.60	4.77
		3	11.35	4.95
		4	11.84	5.06
		5	10.49	5.10
		Total	11.65	5.07
	Males	1	9.32	4.35
		2	9.90	4.19
		3	8.82	3.45
		4	9.90	5.16
		5	9.31	4.86
		Total	9.62	4.75
	Total		10.58	5.06
			11.83	4.76
			10.26	4.51
			11.25	5.17
			10.00	5.02
		Total	10.99	5.06
Phubbing	Females	1	25.56	6.60
		2	25.86	6.50
		3	25.41	5.03
		4	25.42	6.09
		5	23.95	6.52
		Total	25.29	6.30
	Males	1	24.97	6.29
		2	25.39	4.91
		3	23.36	6.62
		4	24.37	6.18
		5	24.52	6.48
		Total	24.59	6.10
	Total	1	25.38	6.50
		2	25.72	6.08
		3	24.52	5.81
		4	25.10	6.13
		5	24.19	6.50
		Total	25.06	6.24

*BIAS* Bergen Instagram Addiction Scale. Education 1—Higher Education, completed; 2 – Higher Education, not completed; 3 – High School, completed; 4- Master’s degree/PhD, not completed; 5 – Master’s degree/PhD, completed

Further tests, e.g., the Bonferroni post-hoc one, showed that, when it comes to the gender factor, only the variable “excessive use of Instagram” presented statistically significant differences, and women (*M* = 11.48; *SD* = 0.21) presented higher scores than men (*M* = 9.45; SD = 0.27, *p* < 0.001).

## Discussion

The aim of this study was to investigate the relationship between phubbing behavior, Instagram use, personality traits based on the Big Five theory, and sociodemographic variables (gender, age, and education). In the present study, the results of the correlation analysis showed that phubbing behavior and excessive use of Instagram are positively correlated with each other. In addition, this behavior showed a positive and moderate correlation with Neuroticism, and negative and weak correlations with age and Conscientiousness. Instagram use had negative and moderate correlations with age; negative and weak correlations with gender; and positive and moderate correlations with Neuroticism. Multiple linear regression analysis (forward method) showed the influence of the variables Neuroticism, Conscientiousness, and excessive use of Instagram on phubbing behavior. MANOVA showed no main effect for education; for gender, only the variable “excessive use of Instagram” showed statistically significant differences, with an average effect size, with women having higher scores than men.

Individuals with high levels of Neuroticism have dysfunctional attitudes towards tasks when they use immature coping strategies to deal with real-life situations, one of which is escaping to OSNs, since these subjects tend to flee from their troubled relationships from real life and spend an excessive amount of time in online environments. They see Instagram as a good option to spend a great deal of time consuming content that seems interesting, i.e., Instagram is used as a positive environment to escape the troubled social relationships of real life (Kircaburun & Griffiths, 2019). In addition, Instagram allows these individuals to hide personal characteristics that are considered undesirable and only show in their profiles what they consider positive. There, they can build a desirable version of themselves through images carefully selected for publication. As such individuals are insecure, they are very likely to lose track of time by reading the comments on their photos and/or videos to find out what other people think and say about them, which may help explain the moderate positive relationship between Instagram Overuse and Neuroticism (Choi et al., 2017; Wiederhold, 2018).

Excessive use of Instagram also showed a positive and strong correlation with phubbing behavior. Individuals who use Instagram excessively are at greater risk of expanding such use to situations where it is considered unwanted or inappropriate, for example, at a family dinner or when meeting friends, thereby disrupting communication. In addition, these individuals tend to be absent-minded while browsing the Instagram feed and fail to realize that time has gone by, and they also have difficulty in balancing the use of the platform. This behavior qualitatively affects the performance of other activities such as work and study, and reduces the time that could be devoted to other important areas of life. This outcome is related to phone obsession, and explains the positive relationship between Instagram use and phubbing behavior (Chen et al., 2017). Furthermore, Van der Schyff et al. (2022) pointed out that this relationship happens because individuals who present an ideal version of themselves on Instagram tend to make excessive use of the network and, consequently, show higher levels of phubbing behavior. This is due to self-presentation through carefully selected and overinflated content, which does not necessarily match reality, but demands a great deal of time to be produced to be able to sustain online selves (Jiang & Ngien, 2020).

People with high levels of neuroticism are unstable and impatient by nature, which can cause them to constantly experience unpleasant emotions, such as a rapid change to an angry state, and face life from a negative perspective (McCrae & John, 1992). Thus, they are more likely to experience troubled social relationships. In this sense, these individuals have hindered communication because they have a poor ability to engage in satisfactory interactions. This is related to the Communication Disorder factor, and it has been confirmed by Çikrikci et et al. (2019), who found a significant correlation between neuroticism and Communication Disorder, which, in turn, explains the relation between neuroticism and phubbing behavior.

In the correlation analysis, Conscientiousness did not show a significant relationship either with phubbing behavior or with Instagram, but the regression analysis showed that Conscientiousness is predictive for such behavior. A negative correlation was found between the Conscientiousness trait and phubbing behavior, that is, more conscientious subjects present such behavior to a less degree, while less conscientious subjects exhibit this behavior more often. Conscientious individuals can control their impulses, have disciplined and patient personalities, can postpone cravings, and manage their time satisfactorily. These subjects will be more aware and cautious about how and how long they use their smartphones. In addition, they behave more responsibly with interpersonal relationships and seek to establish them in more constructive ways, which indicates that their communications will be less disturbed; this works as a protective factor against phubbing behavior, whereas individuals with low conscientiousness are consequently less careful about the quality of their relationships and more likely to have poor discipline and organization (John & Srivastava, 1999; Lee et al., 2019; Mccrae & Costa, 1987; Sleem & El-Sayed, 2011; Živčić-Bećirević et al., 2017).

Low conscientiousness and high neuroticism are implicated in poor emotional behavior when dealing with reality, causing people to escape this unpleasant reality, and making excessive use of OSNs, such as Instagram. Furthermore, they may develop technology-related unhealthy behaviors, e.g., phubbing, which will hinder their social communication and affect the way in which they interact in real life, thus resulting in a paradoxical move.

With regard to gender, it was found that women had higher scores for Instagram use than men. According to Huang and Su (2018), this is because women use Instagram to escape embarrassing situations and boredom more often than men, who generally resort more often to digital games (Ivanova et al., 2020). Another factor that may help explain the more extensive use of Instagram by women is the fact that Instagram is also a business platform. Wally and Koshy (2014), in an exploratory study on Instagram as a marketing tool, found that the platform is widely used by women entrepreneurs—especially for domestic businesses—to make their brands known and get closer to their customers, which also offers physical security and convenience.

## Conclusion

Understanding not only the role of personality in Instagram use and in the development of phubbing behavior among Brazilians, but also the role of Instagram in this behavior, helps researchers narrow down the focus of their studies when addressing the issue of phubbing and the excessive use of Instagram by Brazilians. In particular, Neuroticism and Conscientiousness are the points that require most attention.

In the scientific and academic field, researchers can deepen their understanding of the relationship between neuroticism, conscientiousness, Instagram, and phubbing behavior by focusing on the characteristics of neuroticism and conscientiousness to better understand traits and internal processes that may lead to such behavior. Their findings are expected to support the diagnosis of phubbing and the related interventions.

Since it is known that Instagram use also plays an important role in phubbing behavior, individuals can engage in developing a more moderate use pattern for this social network, for example, only using it when not interacting in a social environment, and by managing the time they spend on it, in order to not interfere with the performance or quality of other important activities. Finally, the present results can contribute to the development of activities, programs and preventive measures aimed at phubbing behavior, focusing on the conscientious use of Instagram, as well as on the aspects related to neuroticism and conscientiousness to encourage the development of higher quality communication skills.

This study had some limitations regarding its methodology; for example, it was carried out by means of self-report tools. Therefore, the results may be affected by biased reports, such as social desirability bias. In addition, the sample was predominantly composed of people with a high level of education, which is not a representative characteristic of the Brazilian population. Even though the present study did not find a significant effect of education on the indices of phubbing behavior and Instagram use, the possible existence of education-related bias should be pointed out, and future studies should consider a normative sample of the Brazilian population.

It is worth mentioning that the correlations between personality, phubbing behavior and Instagram use were moderate and therefore should not be taken as significant. However, they are a good starting point for reflection and understanding of this behavior in the Brazilian context, and further studies are required to investigate the issue more closely.

Ultimately, future studies should consider other variables that may contribute to refine the understanding of the relation between conscientiousness, neuroticism, phubbing behavior, and Instagram use, such as life satisfaction, self-esteem, self-presentation, and social comparison. Furthermore, since this is related to a series of technological addictions, researchers should use brain measurements and investigate the physiological and neurological bases of phubbing behavior.

## Data Availability

The datasets generated and analyzed during the current study are not publicly available due to ethical restrictions but are available from the corresponding author upon reasonable request.

## Abbreviation

OSNs: Online social networks (OSNs)
USA: United States of America
PS: Phubbing Scale
RSPD5: Reduced Scale of Personality Descriptors
BIAS: Bergen Instagram Addiction Scale
BFAS: Bergen Facebook Addiction Scal
ICF: Informed Consent Form
MANOVA: Multivariate Analysis of Variance
